# The impact of lockdown measures during the SARS-CoV-2 pandemic on the management of diabetes in a Northern Gauteng Region of South Africa

**DOI:** 10.11604/pamj.2023.45.129.39255

**Published:** 2023-07-18

**Authors:** Tatenda Nyasha Mujuru, Nelly Petunia Mahlangu, Sekwedi Jackson Ngwetjana, Lasya Christina Bekker, Donald Moshen Tanyanyiwa

**Affiliations:** 1 South African National Bioinformatics Institute, University of the Western Cape, Cape Town, South Africa,; 2 Department of Chemical Pathology, National Health Laboratory Service, Dr. George Mukhari Academic Hospital, Johannesburg, Gauteng, South Africa,; 3 Department of Chemical Pathology, Sefako Makgatho Health Sciences University, Pretoria North, Gauteng Province, South Africa

**Keywords:** SARS-CoV-2, diabetes, hemoglobin A1c (HbA1c), glucose, testing rate (TR), lockdown

## Abstract

**Introduction:**

coronavirus disease 2019 (SARS-CoV-2), a global pandemic, popularised the term “lockdown” due to its rapid spread around the world. “Lockdown” was used as an emergency measure to temporarily prevent people from entering or leaving their communities in an effort to reduce the spread of the virus. The effects of the “lockdown” measures on the management of chronic medical conditions in African populations have been inconsistent. This study aimed to assess the effects of the lockdown on glycaemic control in patients with diabetes.

**Methods:**

retrospective study that examined metadata from 1^st^ January 2019 to 31^st^ December 2021, to assess the impact of the national SARS-CoV-2 response on the quantity and average level of haemoglobin A1c and random glucose in patients with diabetes at Dr. George Mukhari Academic Hospital. The data was retrieved from the National Health Laboratory Services corporate data warehouse.

**Results:**

from 2019 to 2021, a total of 9,039 tests were performed, with females accounting for 63.21% (n = 5,714) and males for 36.08% (n = 3,261), while 0.7% (n = 70) did not have an assigned gender. Mean age was 49, with a standard deviation (SD) of 21.71. The testing rate (TR) in 2019 was 10.74 per day, 2020 had a TR of 6.07, and 2021 had a TR of 7.95. During the pandemic phase, all other age groups had TRs below 1.85, except the 50-59, 60-69, and 70+ year-old groups.

**Conclusion:**

the study revealed that SARS-CoV-2 lockdown measures were linked to poor diabetes control in patients. As a result, the consequences of managing SARS-CoV-2 had a direct influence on diabetes management.

## Introduction

Coronavirus disease 2019 (COVID-19) is a worldwide pandemic that causes severe acute respiratory syndrome with coronavirus 2 (SARS-CoV-2) [1]. Severe acute respiratory syndrome with coronavirus 2 was deemed a global pandemic by the World Health Organization (WHO) in March 2020 [2]. The South African Government initiated a national lockdown on 26^th^ March 2020, with several measures to manage and reduce or contain the rapid spread of SARS-CoV-2 [3]. The public health strategies to reduce the spread of the disease included, among many others, the wearing of facemasks, maintaining social distancing, washing hands more frequently, alcohol and cigarette sale prohibition, and public movement restriction unless seeking or giving medical care [4]. During lockdown alert level 5 (Table 1), worldwide health institutions like Dr. George Mukhari Academic Hospital (DGMAH), in North Pretoria, South Africa, reduced their operations. Medical and hospital activities in Out-Patient Departments (OPDs) like the endocrine and diabetes wards were scaled down, elective surgical procedures were canceled, repeat medication periods were prolonged, and tele-video consultations were introduced. In the initial stages, identification of any single SARS-CoV-2 positive patient resulted in temporary closure for decontamination. Some patients missed their follow-up visits due to the fear of contracting the virus [5]. Coping with pre-existing diseases became difficult due to a lack of access to healthcare facilities and providers [6-8]. This had an impact on routine disease management, particularly in developing countries, which have a higher prevalence of chronic diseases and inadequate health care systems [9].

**Table 1:** lockdown dates as mandated by the South African government

Dates of lockdown	Restriction level	Phase
January 1, 2019 - March 10, 2020	Pre-lockdown	[1] Pre-pandemic
March 11, 2020 - March 25, 2020	No lockdown	[2] Pandemic
March 26, 2020 - April 30, 2020	Alert level 5	[3] Pandemic
May 1, 2020 - May 31, 2020	Alert level 4	
June 1, 2020 - August 17, 2020	Alert level 3	
August 18, 2020 - September 20, 2020	Alert level 2	
September 21, 2020 - December 28, 2020	Alert level 1	
December 29, 2020 - February 28, 2021	Adjusted level 3.1	[4] Pandemic
March 1, 2021 - May 30, 2021	Adjusted level 1.1	
May 31, 2021 - June 15, 2021	Adjusted level 2.1	
June 16, 2021 - June 27, 2021	Adjusted level 3.2	
June 28, 2021 - July 25, 2021	Adjusted level 4	
July 26, 2021 - September 12, 2021	Adjusted level 3.3	
September 13, 2021 - September 30, 2021	Adjusted level 2.2	
October 1, 2021 - December 31, 2021	Adjusted level 1.2	

The currently preferred and commonly used test to monitor and diagnose diabetes is the measurement of glycated haemoglobin (HbA1c) [10]. Also known as haemoglobin A1c, HbA1c results from the glycation of haemoglobin within the red blood cells that is used as a measure for levels of glucose for the past 90-120 days, and the process is non-enzymatic [11]. The periodic monitoring of haemoglobin A1c provides a useful way of documenting the degree of control of glucose metabolism in patients with diabetes [12]. Severe acute respiratory syndrome with coronavirus 2 was a relatively new and understudied disease, but it was observed that comorbidities like chronic health conditions such as diabetes, cardiovascular disease, lung disease, and cancer patients on chemotherapy increased the chances of infection [13]. The wide range of these pathologies resulted in an increased number of laboratory tests, including those with inadequate evidence to justify their use [14]. However, very little research has been conducted to assess the effects of the strategies put in place to control the spread of the virus on comorbidities.

The aim of this study was to assess the effects of the SARS-CoV-2 national lockdown measures on glycaemic control in patients with diabetes that are followed up at the out-patient clinics, peripheral hospitals, and clinics that are serviced by the National Health Laboratory Services (NHLS) Chemical Pathology Department at DGMAH.

The main objective of the study was to compare HbA1c values and random glucose values, as well as their testing rates and/or requests (TRs), during 4 different phases: phase 1 before the SARS-CoV-2 pandemic (pre-pandemic phase); phase 2 during the pandemic but before the national lockdown (no lockdown); phase 3 during the SARS-CoV-2 pandemic national lockdown (alert lockdown levels 5-1); and phase 4 after the revised national lockdown levels (adjusted alert lockdown 1-4) (Table 1). The purpose of this study was to examine the effect of the COVID-19 national response on the amount of TRs for HbA1c and random glucose in patients followed up at OPDs, peripheral hospitals, and clinics that are serviced by the NHLS Chemical Pathology Department at Dr. George Mukhari Academic Laboratory (DGMAL).

## Methods

**Study design and setting:** this was a retrospective study that analysed routine HbA1c laboratory test results metadata for tests conducted at the NHLS DGMAH in the Department of Chemical Pathology from January 2019 to December 2021. Dr. George Mukhari Academic Hospital is one of the largest hospitals in South Africa, located in the north of Pretoria near the township of Ga-Rankuwa. It is a teaching facility for the Sefako Makgatho Health Sciences University (SMU), School of Medicine.

**Study population:** the study was performed on patients of predominantly African descent referred to DGMAH from surrounding district hospitals and clinics in Ga-Rankuwa town, North Pretoria region in the Gauteng province. Results were retrieved from a central database, and therefore, no patient recruitment was required, however, permission was sought from the NHLS data management division. A simple random selection of all patients with diabetes who had their HbA1c and random glucose tested between January 2019 and December 2021 was included. The inclusion criteria were the availability of all demographic, HbA1c, and random glucose results; delta checks confirmed the presence of previous results for patients being followed up at DGMAH. Exclusion criteria were the absence of any results before the SARS-CoV-2 pandemic and missing demographic data.

**Data collection:** data were collected from the NHLS corporate data warehouse (CDW) using a standard data extraction tool and method, Microsoft Excel 2016 (Microsoft Corporation, Redmond, WA). Collected data included the following main variables: HbA1c results, random glucose results, gender, age, and ward. Data clean-up (removal of duplicates and redundancies) was also undertaken before analysis.

**Laboratory analysis:** samples were analysed using standard published analytical methods. Whole blood samples were used to determine HbA1c levels, performed on the Abbott Architect plus immunochemistry analyser (ci8200). The HbA1c assay employs an enzymatic method for the specific measurement of N-terminal fructosyl dipeptides of the β-chain of HbA1c molecules. Pre-treatment of erythrocytes includes lyses during which the haemoglobin is transformed to methaemoglobin by a reaction with sodium nitrate. The addition of Reagent 1 to the sample facilitates cleavage (by the action of protease) of the glycosylated N-terminal dipeptide fructosyl-valyl histidine (VH) of the β-chain of haemoglobin. The haemoglobin is transformed to stable methaemoglobin azide by the action of sodium azide after which the concentration of the haemoglobin is determined by measuring absorbance. Addition of reagent 2 starts a reaction and fructosyl peptide oxidase is allowed to react with fructosyl VH. The HbA1c concentration is measured by determining the resultant hydrogen peroxide [15]. Plasma samples were used to determine glucose concentrations on the Abbott Architect Plus immunochemistry analyser (ci8200) using the hexokinase method. This is an enzymatic method in which glucose is converted to glucose-6-phosphate (G-6-P) by hexokinase in the presence of adenosine tri-phosphate (ATP), a phosphate donor. Glucose-6-phosphate dehydrogenase then converts the G-6-P to gluconate-6-P in the presence of nicotinamide adenine dinucleotide phosphate (NADP^+^). During this reaction, the NADP^+^ is reduced to NADPH (by addition of hydrogen), and the resulting increase in absorbance at 340 nm (secondary wavelength = 700 nm) is measured. This process is known as a glucose-dependent endpoint reaction.

**Definitions:** the indications used to determine the level of restrictions to be applied during the declaration of a state of national emergency by the South African government, and the criteria for the implementation of alert lockdown levels in South Africa was conducted with the objectives summed up as follows: (a) “Alert Level 5”: take drastic efforts to stop the virus's spread and preserve lives - denotes a high SARS-CoV-2 spread with a low health-care system preparedness; (b) “alert level 4”: take extreme care to minimise community transmission and outbreaks while allowing for some activities to resume - denotes a moderate to high SARS-CoV-2 spread with a poor to moderate health system readiness; (c) “alert level 3”: restrictions on activities, including at work and in social settings, to address a high risk of transmission - denotes a moderate SARS-CoV-2 spread with a moderate health system preparedness; (d) “alert level 2”: physical separation and limits on leisure and social activities to prevent the infection from resurfacing - denotes a moderate SARS-CoV-2 spread with a high level of health system readiness; (e) “alert level 1”: most regular activities can continue, with precautions and health recommendations being adhered to at all times. If required, the population should be prepared for an escalation in alert levels - denotes a low SARS-CoV-2 spread with a high level of health system preparation [16]; (f) “adjusted alert levels” were alert levels with certain alterations that were more suited for the economy while not jeopardising the well-being of the community; (g) “pre-lockdown”: the period before SARS-CoV-2 was declared a pandemic; (h) “no lockdown”: the period after SARS-CoV-2 was declared a pandemic but before the national lockdown was imposed. Additional information has been provided indicating detailed lockdown dates in Table 1 [16].

**Statistical analysis:** data were analysed using the statistical analytical method R (R Studio) and Excel (Office 365; Microsoft, USA). These were used to analyse data and construct graphs. Results were grouped and tabulated according to date, lockdown level, and site of collection. The number of requests per analyte was compared between each period of the study, and the mean difference and percentage difference between the phases in question were also determined. The trend of requests for the four distinct phases was also evaluated and compared. Missing data was excluded from all statistical comparison calculations; however, entries with demographic data but only missing a result variable were included.

**Ethical considerations:** ethical approval was granted by the Sefako Makgatho Health Science University Research Ethics Committee (SMUREC) (Reference Number: SMUREC/M/280/2021: IR). The study did not require informed consent from individual participants because there was no need to recruit patients. The data was de-identified before extraction for patient confidentiality and to comply with POPIA regulations. Patient information was accessible only to primary personnel and specific personnel (statisticians and data analysts) who analysed the data.

**Bias:** reliability, validity, and objectivity were ensured by using the recommended cut-off points of HbA1c by the American Diabetes Association (ADA) to classify patients, and participants were naturally selected randomly, which reduced sampling bias. Statistical bias was reduced due to the adequate sample size we received.

## Results

**Figure 1:**
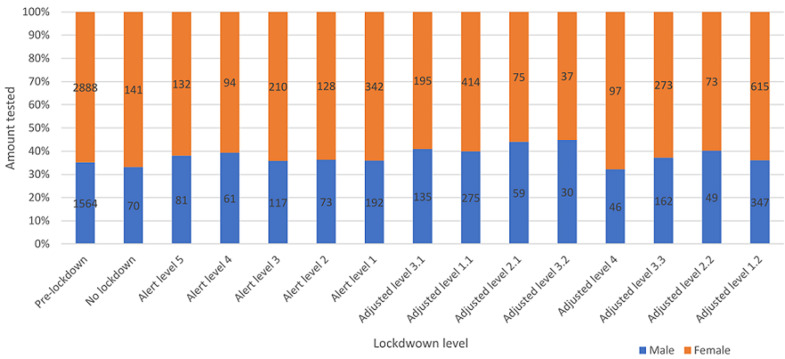
Dr. George Mukhari Academic Laboratory (DGMAL) HbA1c and random glucose test requests between gender

**Patient characteristics:** after removing duplicates and redundancies, a total of 9,039 data entries from January 2019 to December 2021 were retrieved and analysed. The data was then grouped into “no lockdown”, “pre-lockdown”, “alert level lockdown”, and “adjusted alert lockdown level” (Table 1). Females constituted 5,714 (63.21%) of the total, while 3,261 (36.08%) were males, and 64 (0.67%) did not have an assigned gender, giving a male-to-female ratio of 1: 1.75. The ratio of men to women who tested during the “pre-lockdown” level was 1: 1.85, as compared to 1: 2.01 during “no lockdown”, 1: 1.73 during “alert level lockdown”, and 1: 1.61 during “adjusted alert level lockdown” (Figure 1). Females had the single highest frequency across all lockdown levels, with a rate of 31.95% (n = 2,888), which was recorded during the pre-lockdown period. In 2019, the rate of females tested was 27.93% (n = 2,525), which reduced to 15.64% (n = 1,414) in 2020 and then increased to 19.64% (n = 1,775) in 2021. In relation to the males, 15.27% (n = 1,380) were tested in 2019, reducing to 8.62% (n = 779) in 2020, and eventually increasing to 12.19% (n = 1,102) in 2021.

**Figure 2:**
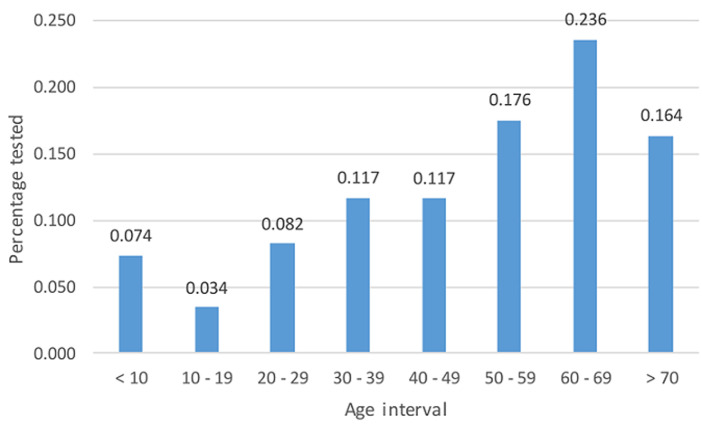
total HbA1c and random glucose amounts tested by age

The highest frequency for males during the pandemic phase was 3.84% (n = 347) during “adjusted alert level 1.2”, while the highest frequency for females was 6.80% (n = 615), also in “adjusted alert level 1.2” (Figure 1). From 2019 to 2021, the average age was 49, with a standard deviation (SD) of 21.71, and 60-69 years old was the group with the most tested patients accounting for 23.6% (n = 2,132) (Figure 2). The mean age for females was 49 with a SD of 21.50, and the mean age for males was 50 with a SD of 21.77. More so, across all lockdown levels, TRs for 60-69 years old were the highest, with TRs as high as 2.48 in “pre-lockdown”. During the pandemic phase, all other age groups had TRs below 1.85, except 50-59 years old during “pre-lockdown” and “no lockdown” (1.89 and 2.53, respectively), 70+ during “pre-lockdown” and “no lockdown” (1.91 and 2.93, respectively), and 60-69 years old during “pre-lockdown”, “no lockdown”, “adjusted alert level 1.1”, “adjusted alert level 2.1”, “adjusted alert level 3.3”, and “adjusted alert level 1.2” (2.48, 3.60, 1.85, 1.88, 1.94, and 2.46, respectively). Nonetheless, of the total, 6,737 entries lacked date of birth (D.O.B.) values and only had age entries, and only three entries lacked both age entries and D.O.B. column entries.

**Figure 3:**
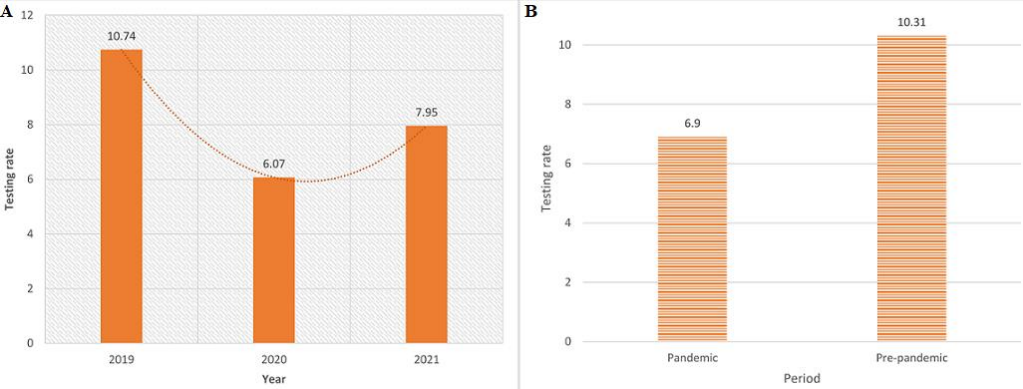
A) Dr. George Mukhari Academic Laboratory (DGMAL) HbA1c testing rates 2019-2021; B) DGMAL HbA1c testing rates before and during COVID-19

**Figure 4:**
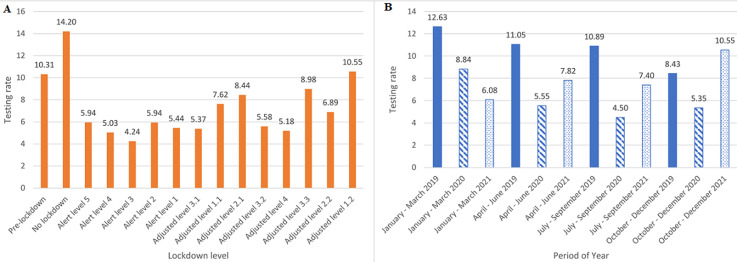
A) Dr. George Mukhari Academic Laboratory (DGMAL) HbA1c and random glucose testing rates between lockdown levels; B) DGMAL HbA1c and random glucose testing rates between yearly quarters

Of the 9,039, 3,921 were tested in 2019 at a testing rate (TR) of 10.74 tests per day, 2,215 in 2020 at a TR of 6.07 tests per day, and 2,903 in 2021 at a TR of 7.95 tests per day (Figure 3A). The pre-pandemic phase (“pre-lockdown”) had the highest average TR of 10.31 tests per day, which reduced during the pandemic phase (“alert lockdown” and “adjusted alert lockdown”) to an average TR of 6.90 tests per day (Figure 3B). “No lockdown” level had the highest average TR of 14.20 tests per day, as compared to “adjusted alert level 1.2”, which had an average TR of 10.55 per day, and a TR of 10.31 was observed in the “pre-lockdown” level. “Alert level 3” had the lowest average TR of 4.24 tests per day, preceded by “alert level 4”, which had an average TR of 5.03 tests per day (Figure 4A). Furthermore, the difference in TRs between the yearly quarters was also analysed, with the highest TRs of 12.63, 11.05, and 10.89 being observed for the periods January to March 2019, April to June 2019, and July to September 2019, respectively. The lowest TR of 4.50 tests per day was observed between July and September 2020, and the TR gradually rises for each period after that, with a sharp increase being observed between the periods of July to September 2021 (7.40 tests per day) and October to December 2021 (10.55 tests per day) (Figure 4B).

**Figure 5:**
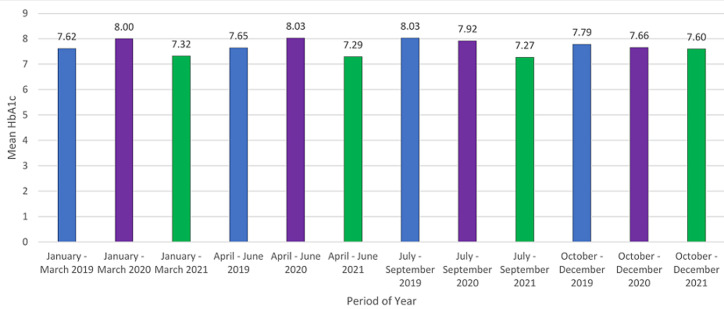
mean HbA1c between yearly quarters, 2019-2021

People with diabetes had the highest frequency across all levels, with the highest frequencies being in the pre-lockdown phase and adjusted level 1 of the lockdown phase. A summary of the association between age and gender in the study population was also conducted, and 60-69 had the highest cumulative frequency in both males (9.6) and females (15.80), with less than 10 years old being the lowest in both genders as well (0.60 and 0.80, respectively). The association between age and the diabetic status of the study subjects was also analysed. People with diabetes aged between 60 and 69 years old had the highest frequency. Of the 9,039 variables, 8,474 had an HbA1c result, with 4,395 having a result greater than 6.5% (people with diabetes), 1,747 having a result between 5.3% and 6.4% (prediabetes), and 2,332 having a result below 5.7% (normal). Of the 8,464 HbA1c results, the highest TR was 13.60 during ‘no lockdown’, followed by 9.92 in “adjusted alert level 1.2”. Of the 9,039 variables, 1,104 had random glucose (RG) results, with 565 having only an RG result (no HbA1c result) and an average random glucose of 6.39 mmol/L. The highest TR was observed to be 1.53 during ‘adjusted alert level 3.3’, followed by 1.35 tests per day during “pre-lockdown”. The mean HbA1c was 7.68% with an SD of 3.30, and the mean random glucose was 6.39 mmol/L with an SD of 6.22. The mean HbA1c and mean random glucose per lockdown level, as well as the mean HbA1c between yearly quarters, were also determined (Table 2, Figure 5).

**Table 2:** mean HbA1c and random glucose results across all lockdown levels

Pandemic phase	Mean HbA1c (%)	Mean glucose (mmol/L)
Pre-pandemic	7.80	6.31
Pandemic	7.57	6.47
**Lockdown level**	**Mean HbA1c**	**Mean glucose**
Pre-lockdown	7.80	6.31
No lockdown	7.77	6.34
Alert level 5	8.32	6.20
Alert level 4	8.19	6.86
Alert level 3	7.80	7.45
Alert level 2	7.95	7.45
Alert level 1	7.67	6.20
Adjusted level 3.1	7.45	5.16
Adjusted level 1.1	7.18	6.40
Adjusted level 2.1	7.28	5.04
Adjusted level 3.2	7.89	4.74
Adjusted level 4	7.77	7.22
Adjusted level 3.3	7.23	7.74
Adjusted level 2.2	6.87	4.93
Adjusted level 1.2	7.60	5.86
**Average mean**	7.68	6.39

HbA1C: hemoglobin A1C

Testing rates between the different wards during different lockdown levels were investigated. The data was collated from a total of 17 wards, which include the diabetic clinic, psychiatric ward, ophthalmology clinic, paediatric, medical, surgical, urology, orthopaedic, gynaecology, and endocrine-OPDs (POPD; MOPD; SOPD; UOPD; OOPD; GOPD; and EOPD, respectively). From 2019 to 2021, the cumulative mean testing rate for all wards was lowest in alert level 3 (total mean = 0.24) and highest in “no lockdown” with a cumulative mean of 0.79. The medical units (MOPD, EOPD, Diabetic, POPD, and GOPD) posted a higher combined cumulative TR of 5.33 than the surgical units (SOPD, UOPD, ophthalmology, cardiology, and OOPD) at 1.41. In the medical units, the diabetic clinic had the highest TR of 4.33 during “no lockdown”, closely followed by MOPD (4.27), also during “no lockdown”. In the surgical units, OOPD had the highest TR of 1.17 during “adjusted alert level 2.2”. Table 3 shows some of our findings.

**Table 3:** association between HbA1c testing rate, ward of test request and lockdown level

Lockdown level	HbA1c testing rates (per day) in wards at DGMAH								
	Diabetic	EOPD	GOPD	MOPD	OOPD	POPD	SOPD	UOPD	Unknown
Pre-lockdown	3.03	0.45	0.61	3.63	0.23	0.34	0.58	0.30	0.43
No lockdown	4.33	1.47	0.53	4.27	0.67	0.13	1.13	0.13	0.53
Alert level 5	2.53	0.56	0.17	1.61	0.14	0.11	0.06	0.11	0.14
Alert level 4	1.39	0.19	0.35	1.81	0.16	0.16	0.19	0.26	0.16
Alert level 3	0.76	0.23	0.23	1.59	0.14	0.17	0.38	0.24	0.27
Alert level 2	1.26	0.35	0.38	2.06	0.38	0.21	0.29	0.24	0.32
Alert level 1	0.55	0.58	0.44	1.96	0.25	0.19	0.44	0.21	0.31
Adjusted level 3.1	0.50	0.15	0.32	2.85	0.10	0.24	0.21	0.26	0.27
Adjusted level 1.1	1.09	0.55	0.60	2.96	0.64	0.25	0.35	0.29	0.48
Adjusted level 2.1	2.06	0.38	0.69	2.94	0.81	0.19	0.31	0.31	0.19
Adjusted level 3.2	1.00	0.08	0.17	2.92	0.33	0.00	0.08	0.50	0.17
Adjusted level 4	0.75	0.39	0.29	2.04	0.25	0.21	0.18	0.43	0.29
Adjusted level 3.3	0.82	0.16	0.49	3.92	0.73	0.27	0.82	0.37	0.55
Adjusted level 2.2	0.28	0.11	0.44	3.00	1.17	0.28	0.22	0.39	0.39
Adjusted level 1.2	2.51	0.57	0.97	3.64	0.83	0.20	0.45	0.50	0.52

HbA1C: hemoglobin A1C, DGMAH: Dr. George Mukhari Academic Hospital (DGMAH), EOPD: Endocrine Out Patient Department, GOPD: Gynaecology Out Patient Department, MOPD: Medical Out Patient Department, OOPD: Orthopaedic Out Patient Department, POPD: Paediatric Out Patient Department, SOPD: surgical Out Patient Department, UOPD: urology Out Patient Department

## Discussion

A greater proportion of females were tested for HbA1c in the ‘pre-lockdown level’ (pre-pandemic stage), and this is expected as previous studies have shown the tendency for females to visit hospitals more than their male counterparts (Figure 2A) [17-19]. Gender stratification of results revealed that during the lockdown period, more females attended the clinics than their male counterparts, with the highest frequency being in the 60-69-year-old female age group. This is likely due to the high prevalence of diabetes in people of older age. An analysis of the different lockdown levels showed that test volumes in “pre-lockdown” were the highest (49.52%; n=4,476), with test volumes in “alert lockdown” (15.95%; n = 1,442) being half of the “adjusted alert lockdown” (32.17%; n = 2,908). Further imposition into the pandemic phase revealed “adjusted alert level 1” to have the highest volume, contributing 18.41% (n=1,664) of the total tested, followed by “adjusted alert level 3”, which was nearly half, recording 9.29% (n=840). The lowest test volumes were observed in “adjusted alert level 4” and “alert level 4”, which contributed 1.60% and 1.73%, respectively. The large test volumes during “adjusted alert levels” can be ascribed to the relaxation of lockdown constraints, with individuals being permitted to move more freely and resume their normal daily activities and business. The low volumes during the alert levels could be attributed to the fact that only extremely ill people were allowed to travel and go to the hospital during the initial parts of the national lockdown.

There were varying mean HbA1c results between the lockdown levels, ranging from 6.87% to 8.32%, with 6.87% being observed in “adjusted alert level 2.2” and 8.32% being recorded during “alert level 5 lockdown”. There were also varying mean random glucose results between the lockdown levels, which ranged between 4.74 mmol/L and 7.74 mmol/L, with 4.74 mmol/L being observed in “adjusted alert level 3.2” and 7.74 mmol/L being recorded in “adjusted alert level 3.3”. The high mean readings could be attributed to the fact that, during the beginning of the COVID-19 pandemic, only extremely ill patients (with extreme diabetic symptoms) would have visited the hospital due to the newly declared pandemic status and newly imposed lockdown restrictions. The low random glucose test turnover is due to the fact that it is usually conducted as a point-of-care test in the hospital and the results are not transmitted to the NHLS.

The TRs of each lockdown level were also analyzed, and the “no lockdown” level was found to be level with the highest average TR of 14.20 tests per day, followed by “pre-lockdown” and “adjusted alert level 1.2”, which recorded 10.31 tests per day and 10.55 tests per day, respectively. These high TRs can be attributed to the ease of lockdown restrictions during these periods. The findings of our study also revealed that the pandemic had a negative impact on the number of patients who attended clinics and the hospital, hence the low rate of testing during this period. Furthermore, our findings show that the rate of HbA1c and random glucose testing was higher before the pandemic (10.67) than during the pandemic (7.27), showing a 31.87% reduction. There was a 42.50% reduction in TR between January and December 2019 and January and December 2020, which was followed by a 30.16% increase in TRs between January and December 2020 and January and December 2021.

Varying studies have shown that during the early months of the pandemic, the number of HbA1c tests performed on outpatients decreased by up to 70% [5,20,21]. The number of HbA1c tests was observed to have declined sharply in April 2020 and gradually increased thereafter globally [21]. In Cape Town, S.A., Kruger and colleagues observed a significant decrease in relevant testing such as HbA1c, particularly in April 2020, when the alert level 5 lockdown was initiated. According to this study, HbA1c testing dropped by approximately 64% between March and June 2020 when compared to the same period the previous year [5].

A WHO study of 37 countries found that 49% had complete or partial disruptions to diabetes and diabetes complication management services, with lower-middle-income countries slightly more likely to report or experience disruptions [2]. The number of HbA1c tests requested decreased rapidly as the pandemic began and progressed. This is consistent with other studies. For example, a study conducted in 47 different countries revealed that diabetes (38%) was the most affected disease globally by the reduction in healthcare resources caused by the pandemic [22]. Fragala *et al*. conducted a study monitoring weekly HbA1c volumes at a large National laboratory situated at Massachusetts General Hospital in Boston, during COVID-19 alert levels (which required people to stay at home) and discovered the test volumes were reduced by 66%. In the first 8 weeks between March 2020 and April 2020, a more significant decline was observed in females (69%) compared to men (62%) [23].

We also observed a significant difference in TR during alert lockdown (16.1%) compared to adjusted lockdown (32.6%). A study on People living with Diabetes (PWD) in Indonesia discovered that 30.1% had difficulty attending diabetes consultations and 12.4% had difficulty accessing diabetes medication. According to their multivariate analysis, during the pandemic, there was a 1.41 increase in diabetic complications [24]. Diabetes was identified as a comorbidity that is a major risk factor for SARS-CoV-2 in a systematic review of six studies involving a total of 1,558 patients [25]. In a retrospective study conducted in Texas, USA, laboratory test volumes showed a decrease in the beginning stages of the state lockdown, which was preceded by semi and fully-completed recoveries of the affected units. Haemoglobin A1c initially declined from 2,232 in April 2019 to 452 in April 2020 which is an 80% reduction. Underuse of testing to treat chronic illnesses, as well as for traditionally marginalized groups and people of colour, was the largest patient hazard during the pandemic [26].

A study by Sharma *et al*. showed that HbA1C testing volumes decreased by 23%, 61%, and 40% in March, April, and May 2020 (respectively) during the pandemic, when compared to the corresponding months in 2019 [20]. In April and May 2020, the frequency of diabetes (HbA1c > 6.4%) increased by approximately 19% before returning to baseline in June [20]. According to this study, for every 1% reduction in testing volume, the frequency of diabetes (HbA1c > 6.4%) increased by 0.3% [20]. The COVID-19 pandemic had an effect on mean HbA1c results [27].

The study´s fortes included the fact that it was retrospective and included a wide dataset over a 3-year period. Furthermore, the study is the first of its kind in southern Africa. Moreover, the study was conducted at the third-largest tertiary health institution in South Africa. Based on the outcomes of this study, we propose that guidelines be established for virtual and telephonic follow-up visits with consultants, as well as point-of-care and home testing. With the advancement of time and technology, healthcare centres and professionals must adopt new methods of healthcare delivery, such as virtual healthcare and digital technologies, in order to continue with routine management [22]. Telehealth has emerged as a valuable service for providing ongoing medical care while reducing the risk of SARS-CoV-2 exposure; more so, telehealth visits can also provide advice and education on diabetes management [28,29]. Telemedicine, which has long been used in other fields such as genetic counselling, has also been shown to be effective in managing HbA1c and random glucose levels and empowering patients [30,31]. Online prescription delivery systems have also been shown to be effective in Asian studies [28,32,33].

## Conclusion

The study revealed that SARS-CoV-2 lockdown measures were associated with poor management of patients living with diabetes. Therefore, the effects of controlling and managing SARS-CoV-2 had a direct impact on the optimum management of diabetes.

### What is known about this topic

- *It was extensively reported that people with chronic conditions like diabetes were at an increased risk of hospitalization, ICU admission, and complications associated with the SARS-CoV-2 infection;*
- *The virus was associated with ill-defined, non-specific pulmonary symptoms that resulted in a wide range of test requests that still left many unanswered questions*.

### What this study adds

- *The study revealed that the lockdown measures implanted across the world contributed to poor glycaemic control in patients living with diabetes; this might have contributed to the increased number of patients presenting with complications of SARS-CoV-2;*
- *The study has initiated the need to explore the consequences of the SARS-CoV-2 infection on the metabolic pathways and organs involved in glucose metabolism*.
